# A Missed Diagnosis of Ovarian Torsion in a Patient with Bilateral Ovarian Dermoid Cysts: A Case Report

**DOI:** 10.7759/cureus.5963

**Published:** 2019-10-22

**Authors:** Agrima Ghosh, Rebecca McKay

**Affiliations:** 1 Surgery, Peterborough City Hospital, Peterborough, GBR; 2 Obstetrics and Gynecology, Peterborough City Hospital, Peterborough, GBR

**Keywords:** dermoid cyst, ovarian, torsion, bilateral, ovarian tumor, diagnosis, ovarian torsion

## Abstract

Ovarian torsion is a gynecological emergency that occurs when there is twisting or torsion of the ovary around its ligamentous supports. If left untreated, it results in loss of blood supply to the ovary and the fallopian tube with resultant infarction and loss of function. Because the presenting symptoms and signs provide a wide differential diagnosis, it can be difficult to diagnose ovarian torsion in the emergency setting. We present a case of a missed diagnosis of ovarian torsion which resulted in the loss of an ovary in a young female with bilateral large ovarian dermoid cysts.

## Introduction

Ovarian torsion is a condition that occurs when there is a partial or complete twisting or torsion of the ovary around its ligamentous supports. If left untreated, it results in interruption of blood and lymphatic supply to the ovary and the fallopian tube with resultant infarction and loss of function.

Ovarian torsion is commonly seen in women of reproductive age but can occur in other age groups as well. The incidence of ovarian torsion ranges from 2% to 5% in patients who have surgical treatment for adnexal masses. The most common adnexal mass associated with ovarian torsion is an ovarian dermoid cyst [1]. A dermoid cyst is a cystic growth, which is usually present at birth and grows slowly over several years. It contains a diversity of tissues including bone, teeth, hair, fluid, and skin. Dermoid cysts commonly occur on the face, within the brain, on the back, and in the ovaries. They can affect one or both ovaries.

Ovarian torsion is a surgical emergency, which requires rapid diagnosis and laparoscopic intervention to ensure preservation of ovarian function. We present an interesting case which also highlights the consequences of missing the diagnosis of ovarian torsion in the emergency setting.

## Case presentation

A 25-year-old lady presented to the emergency department with a two-day history of left iliac fossa pain and vomiting. She was normally fit and well with a history of having had an appendectomy. She did not have any symptoms to suggest urinary tract infection, and she was on her menstrual period during presentation. She did not have any known personal or family history of gynecological problems. She was not on any regular medication.

General examination was unremarkable. Abdominal examination revealed a soft abdomen with tenderness in the suprapubic region and left iliac fossa.

Routine blood results including inflammatory markers were normal (Table 1).

**Table 1:** Patients blood results with laboratory reference values Results shown are from the initial and second presentations

Blood parameters	Normal values	Initial presentation	Second presentation
White cell count (10^9^/L)	4-11	9.2	9.5
Hemoglobin (g/dl)	115-165	140	128
Platelets (10^9^/L)	150-400	203	185
Sodium (mmol/L)	133-146	138	140
Potassium (mmol/L)	3.5-5.3	4.4	3.8
Creatinine (µmol/L)	45-84	68	69
Alkaline phosphatase (U/L)	30-130	61	57
Albumin (g/L)	35-50	49	47
Bilirubin (µmol/L)	<21	17	13
Alanine transferase (U/L)	10-60	14	10
Amylase (U/L)	0-100	71	66
C-reactive protein (mg/L)	<10	<10	<10
Cancer antigen CA125 (U/ML)	<35	57	

**Figure 1:**
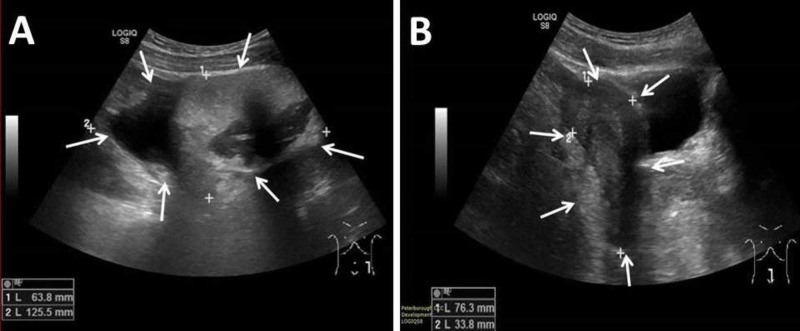
Ultrasound scan of the ovaries A. Left ovary demonstrating a large dermoid cyst, B. Right ovary demonstrating a large dermoid cyst

After the initial review, the patient was sent home with analgesia and to have an outpatient ultrasound scan of the pelvis with transvaginal views. The ultrasound scan, which was performed a month later, revealed a mixed echogenic mass measuring 12.6 cm x 8.5 cm x 6.4 cm within the left adnexa. Within the right adnexa, a similar appearing mass was also demonstrated measuring 6.9 cm x 6.1 cm x 4.1 cm. No free fluid was seen in the pelvis. The appearance was consistent with bilateral ovarian dermoid cysts (Figure 1).

At a three-month clinical review, a magnetic resonance imaging (MRI) scan was booked. However, she was admitted to the emergency department again with left iliac fossa pain and vomiting. Abdominal examination was unremarkable, and routine blood tests were normal. The plan was then made to send her home and wait for the MRI scan on an outpatient basis, and if the pain worsened in the interim, to have emergency surgery to remove the cysts.

**Figure 2:**
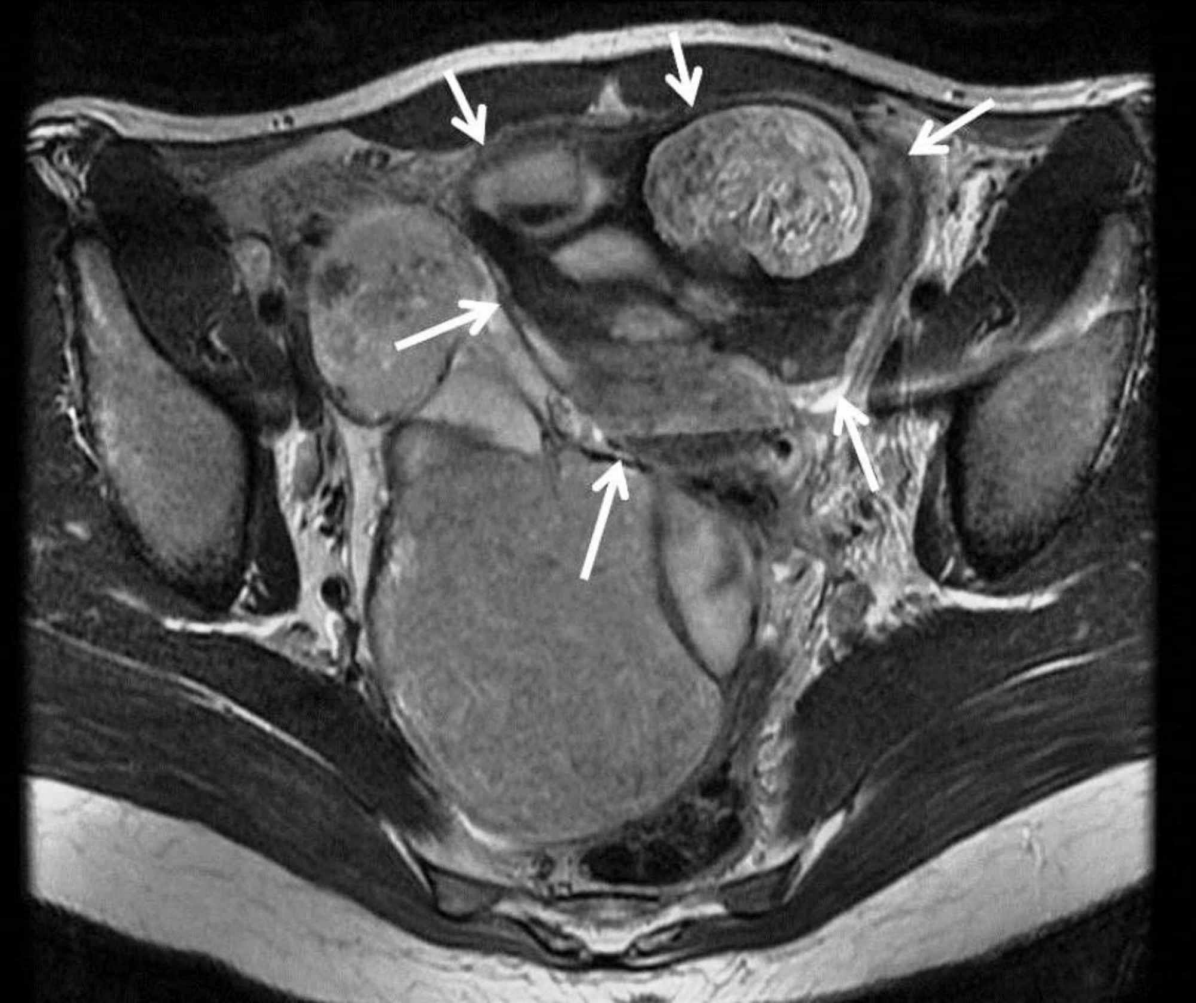
Magnetic resonance imaging scan of the pelvis View demonstrating a large thin-walled dermoid cyst of the left ovary

The MRI scan revealed large bilateral adnexal masses, measuring up to 16.6 cm x 13.7 cm x 9.1 cm on the right side and 13.2 cm x 10.7 cm x 8.2 cm on the left side. The right-sided lesion occupied most of the right hemi-pelvis and descended between the uterus and rectum caudally. The left-sided lesion was located between the uterus and anterior abdominal wall extending cranially and occupying partially the left upper quadrant of the abdomen. Both lesions were complex with several components, some cystic and some containing large amount of fat in keeping with dermoid cysts or teratomas (Figure 2).

After discussing the risk of surgery and possible effects on future fertility, the patient underwent laparoscopic surgery two months later. This revealed two large bilateral ovarian cysts filling the pelvis. The right ovary had multiple dermoid cysts, which were removed piecemeal sparing a large remnant of the right ovary together with a normal right fallopian tube. The left ovary appeared inflamed and gangrenous, and it was surrounded by omental adhesions, most likely due to chronic torsion. Removal of the omental adhesions exposed the fact that the left ovary had undergone torsion twice, and it was felt that this ovary should be removed. There were no intra-operative complications. The majority of the operation was undertaken laparoscopically although the large cyst was taken out through a small Pfannenstiel incision with plenty of pelvic wash out.

Histology confirmed bilateral large benign dermoid cysts. There was also evidence of focal infarction of the left ovary in keeping with ovarian torsion.

**Figure 3:**
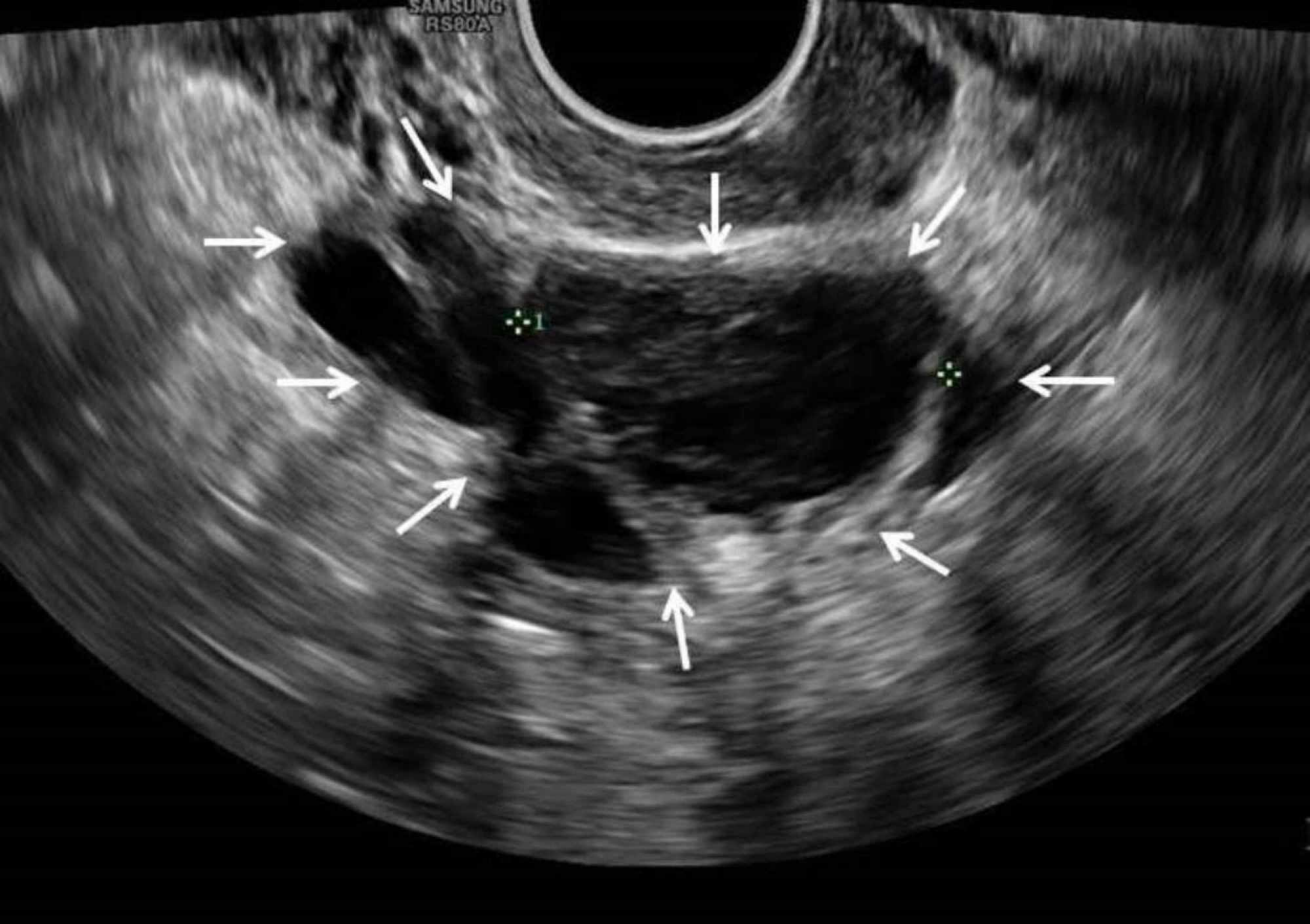
Ultrasound scan of the pelvis Demonstrating a small right ovarian cyst measuring 3.5 cm with internal echoes and stringy septa

She was followed up in the gynecology clinic two months later. She was doing very well and had two normal menstrual periods since the operation. She had no more pain or discomfort. However, her level of anti-Mullerian hormone level becomes low at 1.8 pmol/L indicating very low ovarian reserve (very low fertility potential) in the remaining right ovary. A repeat transvaginal ultrasound scan revealed a right ovary with small cysts (Figure *3*).

## Discussion

Ovarian torsion is a gynecological emergency due to a complete or partial rotation of the adnexal supporting organ, resulting in ischemic changes in the ovary. Torsions more commonly involve both the ovary and fallopian tube, and there are fewer cases of isolated torsions involving just the ovary or fallopian tube. The primary risk factor for an ovarian torsion is an ovarian mass. The size of the ovarian mass has a positive correlation with the risk of torsion. Ovarian tumors larger than 5 cm carry a high risk of ovarian torsion [1]. 

The clinical presentation is often non-specific with abdominal pain, nausea, and vomiting. Clinical examination may reveal a pelvic mass and tenderness. Initial investigations should include doing a full blood count, electrolytes, and a pregnancy test. Serum human chorionic gonadotropin and cancer antigen 125 (CA125) can be used to detect ovarian germ cell tumors and malignant tumors, respectively. Other useful tumor markers include carcinoembryonic antigen, alpha-fetoprotein, and lactic dehydrogenase [1,2]. Elevated tumor markers must be interpreted in context as slightly raised CA125 levels are also associated with non-malignant gynecological diseases, as in this case [3].

A transvaginal and abdominal ultrasound with central venous Doppler studies should be the first-line investigation for looking for adnexal mass and decreased or absent vascular flow in the ovarian vessels. MRI is helpful in diagnosing ovarian torsion if findings on ultrasound scanning are equivocal [1]. Laparoscopy or laparotomy is required for definitive diagnosis and for careful thorough inspection and examination of the ovaries for viability [1].

Despite the tell-tale signs, a diagnosis of ovarian torsion is still a challenge in the emergency setting. Symptoms and signs are variable, the differential diagnosis is wide, sonographic features are inconsistent, and ovarian salvage is rare [4-6]. The occurrence of the characteristic symptoms (abdominal pain, nausea, and vomiting) in a patient with an ovarian cyst greater than 5 cm in size combined with a high clinical awareness and suspicion remains the key feature available to enable early diagnosis and management [4-6].

The patient is this case had two emergency attendances with left iliac fossa pain, nausea, and vomiting. She was also found to have bilateral large ovarian cysts on ultrasound scan and an MRI scan. When she finally had surgery to remove the cysts, an unsalvageable left ovary due to double torsion was discovered. This is an expected outcome, which comes with a delay in diagnosis of ovarian torsion [7]. The importance of early diagnosis and management cannot be overemphasized. There was failure to recognize ovarian torsion as a differential diagnosis during the initial and subsequent presentation of this case, despite the ultrasound findings in between the two presentations. 

There are a number of lessons that can be learnt from this case report. First, a combination of symptoms of abdominal pain (especially in the iliac fossa region), nausea, and vomiting in a female requires urgent gynecological assessment. Second, such patients require urgent imaging by an experienced sonographer (bedside sonography would be desirable), especially if there is a palpable, tender pelvic mass (waiting for an MRI scan to be performed may delay surgical management). Third, the discovery of a painful ovarian or adnexa mass or cyst should arouse the suspicion of an ovarian torsion, which is a gynecological emergency. Fourth, any subsequent presentation with pain should be managed as a surgical emergency. 

## Conclusions

We have presented a case of missed diagnosis of ovarian torsion which resulted in the loss of an ovary in a young female with bilateral large ovarian dermoid cysts. This case highlights the importance of considering ovarian torsion in young women presenting to the emergency department with acute abdominal pain, and the need for prompt gynecology review and imaging.
